# Rapid radiation of ant parasitic butterflies during the Miocene aridification of Africa

**DOI:** 10.1002/ece3.10046

**Published:** 2023-05-13

**Authors:** Marianne Espeland, Nicolas Chazot, Fabien L. Condamine, Alan R. Lemmon, Emily Moriarty Lemmon, Ernest Pringle, Alan Heath, Steve Collins, Wilson Tiren, Martha Mutiso, David C. Lees, Stewart Fisher, Raymond Murphy, Stephen Woodhall, Robert Tropek, Svenja S. Ahlborn, Kevin Cockburn, Jeremy Dobson, Thierry Bouyer, Zofia A. Kaliszewska, Christopher C. M. Baker, Gerard Talavera, Roger Vila, Alan J. Gardiner, Mark Williams, Dino J. Martins, Szabolcs Sáfián, David A. Edge, Naomi E. Pierce

**Affiliations:** ^1^ Centre for Taxonomy and Morphology Leibniz Institute for the Analysis of Evolutionary Change – Museum Koenig Bonn Germany; ^2^ Department of Organismic and Evolutionary Biology and Museum of Comparative Zoology Harvard University Cambridge Massachusetts USA; ^3^ Department of Ecology Swedish University of Agricultural Sciences Uppsala Sweden; ^4^ CNRS UMR 5554 Institut des Sciences de l'Evolution de Montpellier Montpellier France; ^5^ Department of Scientific Computing Florida State University Tallahassee Florida USA; ^6^ Department of Biological Science Florida State University Tallahassee Florida USA; ^7^ Lepidopterists' Society of Africa Knysna South Africa; ^8^ African Butterfly Research Institute Nairobi Kenya; ^9^ Nature Kenya Nairobi Kenya; ^10^ Department of Life Sciences Natural History Museum London UK; ^11^ Mzuzu Malawi; ^12^ Department of Ecology, Faculty of Science Charles University Prague Czechia; ^13^ Institute of Entomology, Biology Centre Czech Academy of Sciences Ceske Budejovice Czechia; ^14^ Chênée Belgium; ^15^ Institut Botànic de Barcelona (IBB, CSIC‐Ajuntament de Barcelona) Barcelona Spain; ^16^ Institut de Biologia Evolutiva (CSIC‐UPF) Barcelona Spain; ^17^ Southern African Wildlife College Hoedspruit South Africa; ^18^ Turkana Basin Institute Stony Brook University Stony Brook New York USA; ^19^ Institute of Silviculture and Forest Protection University of Sopron Sopron Hungary

**Keywords:** butterfly–ant interactions, *Lepidochrysops*, Lycaenidae, myrmecophagy, phytopredation

## Abstract

Africa has undergone a progressive aridification during the last 20 My that presumably impacted organisms and fostered the evolution of life history adaptations. We test the hypothesis that shift to living in ant nests and feeding on ant brood by larvae of phyto‐predaceous *Lepidochrysops* butterflies was an adaptive response to the aridification of Africa that facilitated the subsequent radiation of butterflies in this genus. Using anchored hybrid enrichment we constructed a time‐calibrated phylogeny for *Lepidochrysops* and its closest, non‐parasitic relatives in the *Euchrysops* section (Poloyommatini). We estimated ancestral areas across the phylogeny with process‐based biogeographical models and diversification rates relying on time‐variable and clade‐heterogeneous birth‐death models. The *Euchrysops* section originated with the emerging Miombo woodlands about 22 million years ago (Mya) and spread to drier biomes as they became available in the late Miocene. The diversification of the non‐parasitic lineages decreased as aridification intensified around 10 Mya, culminating in diversity decline. In contrast, the diversification of the phyto‐predaceous *Lepidochrysops* lineage proceeded rapidly from about 6.5 Mya when this unusual life history likely first evolved. The Miombo woodlands were the cradle for diversification of the *Euchrysops* section, and our findings are consistent with the hypothesis that aridification during the Miocene selected for a phyto‐predaceous life history in species of *Lepidochrysops*, with ant nests likely providing caterpillars a safe refuge from fire and a source of food when vegetation was scarce.

## Graphical abstract

Africa has undergone a progressive aridification during the last 20 My that presumably impacted organisms and fostered the evolution of life history adaptations. Using anchored hybrid enrichment we constructed a time‐calibrated phylogeny for *Lepidochrysops* and its closest, non‐parasitic relatives in the *Euchrysops* section (Poloyommatini, Lycaenidae) and performed ancestral area analysis and diversification analyses. We found that Tte Miombo woodlands were the cradle for diversification of the *Euchrysops* section, and our findings are consistent with the hypothesis that aridification during the Miocene selected for a phyto‐predaceous life history in species of *Lepidochrysops*, with ant nests likely providing caterpillars a safe refuge from fire and a source of food when vegetation was scarce.

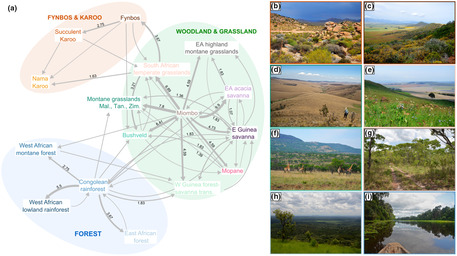

## 1 INTRODUCTION

Africa has undergone dramatic climatic changes since the Eocene (Axelrod & Raven, 1978). From being largely covered by tropical forests, the landmass underwent cycles of drier and wetter climates and experienced a much more dramatic loss of forest cover than other continents, especially during the last 10 million years (Kissling et al., 2012). The combination of tectonic uplift (Jung et al., 2014; Sepulchre et al., 2006), expansion of the polar ice caps, decline in global temperatures (Zachos et al., 2001), shrinkage of the Tethys Sea (Zhang et al., 2014), and changes in oceanic circulation (Cane & Molnar, 2001; Haug & Tiedemann, 1998; Marlow et al., 2000) led to increasing climatic variability and aridification from the late Miocene onwards. As it got drier, grasses became more common, leading to increased grazing and more frequent fires, which further amplified aridification. The modern savanna and grassland biomes now dominating much of the continent did not become fully established until between 8 and 3 Mya in the late Miocene and Pliocene (Edwards et al., 2010; Strömberg, 2011).

The increasing aridification had a strong impact on the organisms inhabiting these areas and on their diversification. This effect has primarily been studied in plants of the Greater Cape floristic region (GCFR), which includes mainly summer arid fynbos, succulent and Nama karoo biomes (Born et al., 2007). This area is a model system for studies of plant diversification because of its remarkably high plant species diversity and endemism. Despite their floral diversity, the current GCFR biomes originated only within the last 10 Myrs (fynbos), and the driest parts (succulent karoo) originated <5 Mya (deMenocal, 2004; Feakins & deMenocal, 2010; Linder, 2003). This aridification is hypothesized to have led to widespread extinction of earlier flora, opening up niches for the diverse communities seen today, dominated by lineages pre‐adapted to an arid climate (Verboom et al., 2009).

In contrast to the flora, the insect diversity in the GCFR, in general, is not unusually high, with numbers comparable with that of neighboring biomes (Giliomee, 2003; Procheş & Cowling, 2006). Ant richness is not particularly high (Braschler et al., 2012), and the number of butterfly lineages is extremely underrepresented (Cottrell, 1985). On these grounds, mechanisms leading to high plant diversity are not thought to have had a strong influence on insects (Braschler et al., 2012). There are, however, indications of co‐divergence between plants and their pollinating flies in the GCFR (de Jager & Ellis, 2017), and similarly, the diversity of pollinating bees is high (Kuhlmann, 2009). Elsewhere in Africa, studies have concentrated largely on forest insects, which originated well before the Miocene when forests were still extensive, and/or diversified during the retreat and isolation of forests from the Miocene towards the present (Aduse‐Poku et al., 2009, 2021; Eberle et al., 2017; Sahoo et al., 2018). Two notable exceptions are ant parasitic beetles (Carabidae: *Paussus*), which radiated extensively also in drier areas in the Afrotropical region within the last 20 million years (Moore & Robertson, 2014), and ant‐associated butterflies such as the lycaenid genera *Chrysoritis*, *Aloeides*, and *Thestor*, with a moderately large number of species in lineages found largely in Southern Africa (Heath et al., 2023; Quek et al., 2022; Rand et al., 2000; Talavera et al., 2020). This suggests that ant association may have played an important role in the persistence and diversification of these insects, but how exactly ant association, and especially parasitism, might have influenced species diversification is not known.

While more than 99% of butterfly larvae feed on plants, entomophagy (obligately feeding on other insects or their secretions, and in the case of ant parasitism, feeding on the brood directly or being fed by worker ants like cuckoos) has evolved many times independently in Lycaenidae. It occurs in at least 31 genera in four of the currently recognized subfamilies. Despite originating repeatedly, this life history has been largely viewed as an evolutionary dead‐end because of its tippy distribution (Cottrell, 1984; Pierce, 1995; Pierce et al., 2002; Schär et al., 2018). Phyto‐predation involves a particularly unusual form of entomophagy in which obligate plant associations have also been retained: caterpillars initially feed on flower buds, but later switch to preying on ants. Phyto‐predation has evolved independently at least twice in the butterflies: once in the Palaearctic genus *Phengari*s (=*Maculinea*, 11 described species) and once in the Afrotropical genus *Lepidochrysops* (137 described species) (Cottrell, 1984; Vila et al., 2011). The main differences between these two lineages are the ant hosts—species of Myrmicinae in *Phengari*s and species of Formicinae in *Lepidochrysops*—and the time of entry into the ant nest, fourth instar in *Phengaris* and third instar in *Lepidochrysops*, where known. Caterpillars of species from both genera can be attacked by specialized parasitoids before and after entering the ant nests (Claassens, 1976; Elgar et al., 2016; Thomas & Elmes, 1993), so entering nests is likely not simply a result of acquiring enemy‐free space. Rather, harsh conditions above ground such as fire or dry seasons, are hypothesized to have triggered shifts into ant nests (Cottrell, 1984; Fiedler, 1991, 1998).

Understanding the origin and evolution of phyto‐predation in *Lepidochrysops* requires examination of character evolution across this genus and its relatives in the *Euchrysops* section sensu Eliot (1974) (Lycaenidae, Polyommatinae). This clade provides an excellent system to examine how life histories have been affected by the aridification of Africa. It contains approximately 210 fully Afrotropical species in five genera, with one exception occurring from India to Fiji (Williams, 2020). Species occur in all habitats from rainforest to semi‐deserts and show various levels of ant association from nearly none (*Thermoniphas*) to facultative and obligate mutualism (*Euchrysops*, *Orachrysops*, *Oboronia*) to obligate phyto‐predation (*Lepidochrysops*) (Cottrell, 1984; Fiedler, 1991; Pierce, 1995; Williams, 2020). Different relationships between the genera have been hypothesized based on morphological and ecological data, and the monophyly of genera has been questioned (Edge & Van Hamburg, 2010; Libert, 2001), but the section has never been included in molecular studies.

## 2 MATERIALS AND METHODS

### 2.1 Taxon sampling

A total of 179 samples, representing 124 species of the *Euchrysops* section and seven outgroups, were included in our study. This includes 80 of 137 described *Lepidochrysops* species, nine of 11 *Orachrysops* species, 14 of 28 *Euchrysops* species, six of seven *Oboronia* species, and seven of 15 *Thermoniphas* species. Many of the missing species are very scarce in collections or only known from the type locality. A specimen of *Lycaena phlaeas* (Lycaeninae) was included to root the trees. Sample information can be found in Data S1B.

### 2.2 Probe design

We developed enrichment probes targeting 200 Anchored Hybrid Enrichment (AHE) loci (Lemmon et al., 2012), 400 anonymous loci, and four legacy loci. We used the following genomic resources: (1) previously published assembled genomes (*Danaus plexippus* [Zhan et al., 2011] *Heliconius melpomene* [The Heliconius Genome Consortium, 2012]); and (2) ~20× coverage raw genomic reads from Espeland et al. (2018) (*Phengaris arion*, *Lepidochrysops patricia*) and this study (*Euchrysops cnejus*, *Jalmenus evagoras*) (Data S1C). We prepared libraries from DNA extracts of the four latter species (following Prum et al., 2015) and sequenced the libraries with a paired‐end 100 bp protocol (single, 8 bp indexing) on Illumina HiSeq2000 sequencers at the College of Medicine Transitional Lab at Florida State University and at the Hudson Alpha Institute for Biotechnology. More information about these genomic resources is available in Data S4.

#### 2.2.1 Selection of AHE target loci

The Lepidoptera‐wide AHE probe design developed by Breinholt et al. (2018) targets 855 exons. We used the six genomic resources described above to increase the size of the target regions and to increase the representation of Lycaenidae. After merging overlapping reads following (Rokyta et al., 2012), we mapped the merged reads to the *Bombyx mori* probe region sequences of Breinholt et al. (2018). The consensus sequence of the mapped reads at each locus for each species was then used as references that were extended 1000 bp further into each flank (see Hamilton et al., 2016 for details). The two assembled genomes were scanned for the presence of the 855 *B. mori* AHE sequences and a 2000 bp region containing each AHE locus was extracted. For each locus, we aligned the six resulting sequences using MAFFT (v7.023b1; Katoh & Standley, 2013). Alignments were inspected in Geneious R9 (Kearse et al., 2012), then trimmed and masked to remove regions that were poorly aligned, poorly represented by the six species, or potential paralogs. This process resulted in 496 candidate AHE targets. Of these, 22 additional targets were removed because they were overlapping and 274 were found too short (<290 bp). The final AHE target set contained 200 loci, averaging 745 bp.

#### 2.2.2 Selection of anonymous target loci

To ensure the resolution of shallow‐scale relationships, we also developed anonymous loci following (Banker et al., 2020). We profiled 30‐mers from the *E. cnejus* and *L. patricia* merged reads to estimate copy number and selected 10,000 reads (per species) with less than 100‐fold read coverage. Using 45‐mers to establish a match, we extended these reads up to 2000 bp in each direction using the remainder of the reads (Hamilton et al., 2016). Only loci with the length between 500 bp and 4000 bp, GC content between 32% and 42%, and average coverage between 18 and 35 (approximate range expected for single‐copy genes given the sequencing effort) were kept, leading to 2790 *E. cnejus* and 2078 *L. patricia* candidate loci. To ensure that targets would work across Lycaenidae, we mapped merged reads from each species to the candidate locus sequences of the other. We extended the consensus of the mapped reads 2000 bp in each direction, then aligned the corresponding sequences for the two species using MAFFT (v7.023b). After selecting the best 1200‐bp region in Geneious, we selected 400 loci at random.

#### 2.2.3 Incorporation of legacy loci

We also incorporated four loci that have been frequently used in other studies into the target set: cad, elongation factor 1 alpha, histone 3, and wingless. Alignments for these genes were obtained from GenBank and subsampled taxonomically to contain only four or five species representing the diversity of Lycaenidae. The four resulting alignments contained 745, 1171, 328, and 403 bp respectively.

#### 2.2.4 Probe generation

We identified and masked repetitive regions in the alignments, following (Hamilton et al., 2016). Probes were tiled uniformly at 4× density across the six taxa in each alignment. A total of 52,749 probes covered a target size of 631,529 bp. A key showing how the target loci correspond to the kit (Breinholt et al., 2018) and locus type can be found in Data S1D.

### 2.3 Molecular methods, data cleaning, and assembly

DNA was extracted from ethanol‐preserved thorax or leg tissue, or dried legs using either the Qiagen Blood & Tissue kit or an AutoGenPrep 965 Tissue DNA Extraction Kit (Autogen). DNA concentration was measured using a Qubit dsDNA HS or BR Assay kit on a Qubit 2.0 fluorometer (ThermoFisher Scientific). We prepared dual‐indexed Illumina libraries following (Meyer & Kircher, 2010), with adaptations in Prum et al. (2015). In short, we sonicated extracted DNA using a Covaris ultrasonicator to a size of ~300 bp. After adding adapters using a Beckman Coulter FxP liquid‐handling robot, we quantified, then pooled (in equal concentration) libraries into 16‐sample pools. We enriched these library pools using the AHE probes described above (Agilent SureSelect XT probes), then quantified and pooled the resulting enriched library pools for sequencing. We sequenced the libraries on 11 Illumina HiSeq2500 PE150 lanes (~450 Gb in raw data). Molecular work was carried out at Harvard University and Florida State University.

After demultiplexing raw reads using 8 bp dual indexes (no mismatches allowed), we removed adapters, corrected for sequencing errors, and merged overlapping reads following (Rokyta et al., 2012). We assembled the reads using sequences from all five probe‐design species (see above) as divergent references in a quasi‐denovo reference assembly (as described in Hamilton et al., 2016). Assembly clusters containing fewer than 100 reads were removed to prevent any contaminated samples from being used downstream. We constructed a consensus sequence for each assembly cluster by statistically distinguishing between sequencing error and heterozygosity (Hamilton et al., 2016). Haplotypes were phased following (Pyron et al., 2016). We established orthology among homologous consensus sequences using alignment‐free pairwise distances and a neighbor‐joining approach, as outlined in Hamilton et al. (2016). After aligning the corresponding orthologous haplotype sequences using MAFFT, we trimmed and masked the misaligned regions following (Hamilton et al., 2016), but with masking parameters set to MINGOODSITES = 15, MINPROPSAME = 0.5 and MISSINGALLOWED = 0.5. We visually inspected alignments in Geneious to ensure that the automated masker and trimmer settings were appropriate.

### 2.4 Molecular data, phylogeny, and dating

The final dataset contained 179 taxa, 419 loci (196 anchored and 223 anonymous loci), and 256,998 bp (average locus length, 613 bp), with 14.1% missing data. Summary statistics for each locus alignment were calculated using AMAS (Borowiec, 2016) (Data S1A). In the concatenation approach, one allele for each taxon was chosen randomly following (Barrow et al., 2014). Trees were inferred with IQ‐TREE 1.6.7 (Nguyen et al., 2015), using ModelFinder (Kalyaanamoorthy et al., 2017) for finding the best partition scheme (greedy algorithm) and model selection. Alignments and the selected models for each partition can be found on Zenodo (DOI: 10.5281/zenodo.4590738). Ten likelihood searches were performed and the tree with the highest likelihood was selected. Branch support was calculated using ultrafast bootstrap support (Hoang et al., 2018) with the ‐bnni option to reduce the risk of overestimating support, and using the SH‐like approximate likelihood ratio test (Guindon et al., 2010), both with 1000 replicates. A species tree was inferred in ASTRAL‐III (Zhang et al., 2017) with the method by Rabiee et al. (2019) for multi‐allele data. Gene trees were generated for each locus based on phased data in IQ‐TREE, as above, and used as input. Local posterior probabilities (LPP) (Sayyari & Mirarab, 2016) were calculated as a measure of support. Molecular dating was performed with a reduced, unphased, unpartitioned, dataset with one representative per species (124 spp.) in MCMCtree 4.9g (Yang, 2007) using approximate likelihood estimation and an independent clock. The topology obtained from the concatenation analyses above, reduced to only include one member per species, was used as fixed input topology. Model selection on this unpartitioned dataset was run in ModelFinder as above, but only allowing the models available in MCMCtree, and the selected model (HKY85) was used in dating analyses. No fossils are available for the family Lycaenidae, and we, therefore, used secondary calibrations from a recent, dated genus‐level butterfly tree (Chazot et al., 2019). Based on that study, we set the split between *Euchrysops* and *Lepidochrysops* as a uniform prior with a lower bound of 9 My and an upper bound of 21 My. Finally, the root calibration was set as a uniform prior with lower bound of 44 My and an upper bound of 69 My based on the age of Lycaeninae + Theclinae + Polyommatinae from Chazot et al. (2019). Other priors were kept as default. We ran four runs with a burn‐in of 100,000, sample frequency of 1000 and number of samples set to 10,000. Convergence was assessed in Tracer 1.7.1 (Rambaut et al., 2018) and by plotting the mean ages and the 95% highest posterior density (HPD) credibility intervals from the posterior distribution of all four runs against each other. Plots showing convergence of runs are available on Zenodo (DOI: 10.5281/zenodo.4590738). The dated tree with credibility intervals (ci) can be found in Figure S3.

### 2.5 Historical biogeography

**FIGURE 1:**
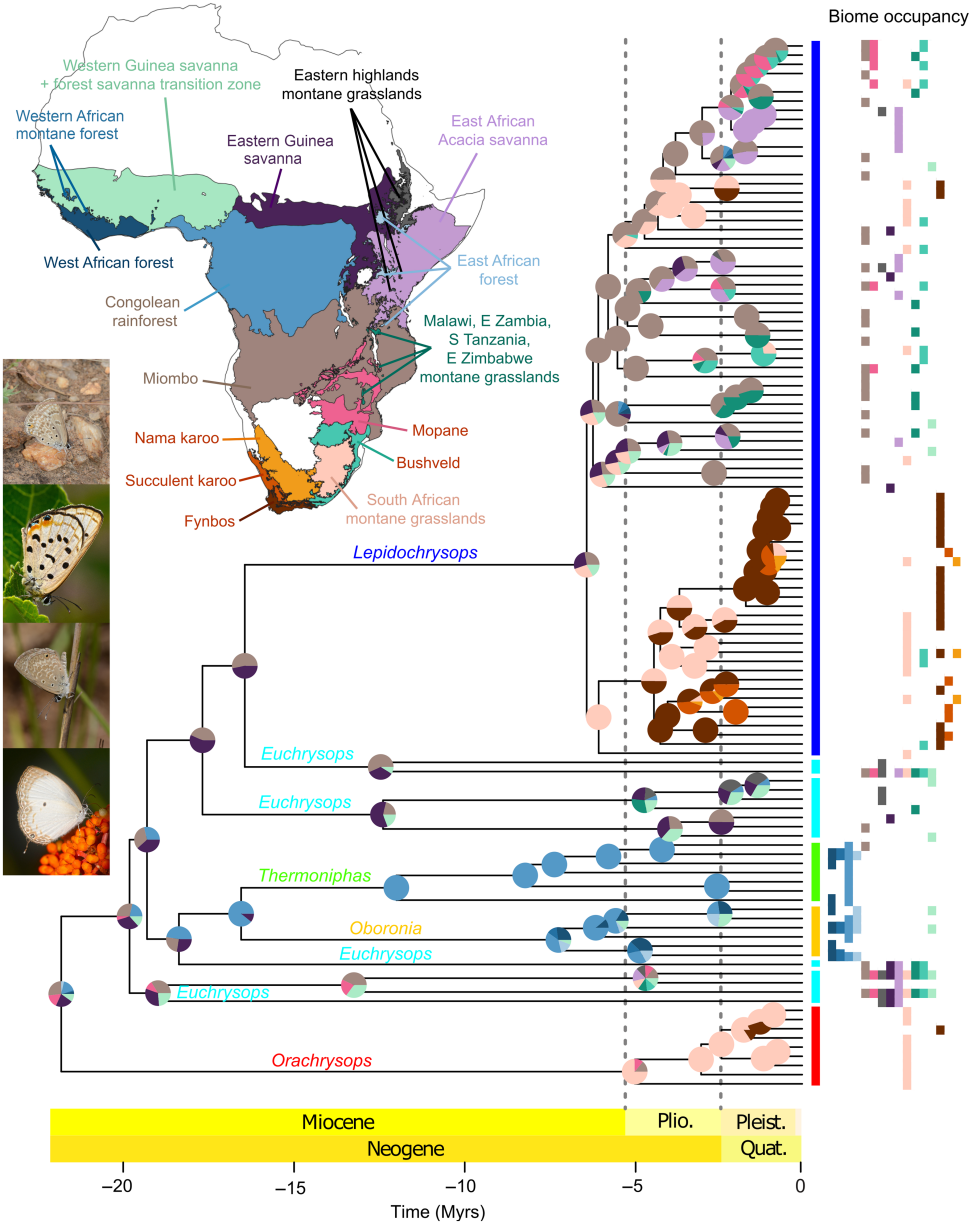
Dated phylogeny of the *Euchrysops* section from MCMCtree with results from the biogeographical analysis as pie charts on the nodes. Colored boxes to the right of the tree show the biomes currently occupied by the included extant species as shown on the map. The various montane grasslands and the West African montane forests are scattered within other biomes, and only partially visible on the map. Pictured butterflies are from top to bottom: *Lepidochrysops abyssiniensis loveni*, *Lepidochrysops peculiaris*, *Euchrysops subpallida*, and *Oboronia punctatus*. All photos by M. Espeland.

We inferred ancestral areas using DECX (Beeravolu & Condamine, 2016), a C++ implementation of the dispersal‐extinction‐cladogenesis (DEC) model (Ree & Smith, 2008). Biogeographical areas were defined based on a simplified classification of the ecosystems of Africa by Dinerstein et al. (2017), including a total of 16 biomes (Figure 1, Data S1E). The map showing these biomes was produced using QGIS v. 3.14 (QGIS Development Team, 2020). The single included species from Madagascar, *Lepidochrysops* cf. *azureus*, as well as the only non‐Afrotropical species, *Euchrysops cnejus*, were both excluded from the biogeographical analyses to reduce the number of biomes. Similarly, *Oboronia bueronica*, the only species found in the East African coastal forests, which is a separate biome, was added to the “East African forest” category. We designed a time‐stratified model in which areas and ranges possibly occupied varied across four different time periods: 0–5, 5–8, 8–11, and 11–25 Mya (Data S1F). The maximum ancestral range size was set to four areas.

Ancestral area estimation was used to calculate the relative frequency of lineages in large biome categories through time. We first identified branches along which dispersal occurred by comparing the ranges with the highest probability between ancestral and descendent nodes. When a dispersal event was detected, we assigned the branch mid‐point as the timing for the dispersal event. Time was then divided into 0.5 My intervals, and within each interval, the number and relative frequency of lineages in each biome was calculated. Rather than working with the 16 areas, we combined them into the following functional categories: forests, woodland and grassland, and Fynbos and Karoo. Within each category we also separated the lineages belonging to *Lepidochrysops* from the rest of the tree (backbone), to calculate, for example, the frequency of forest species versus non‐forest species within each part of the tree.

We also compared the number of transitions between biomes. We identified branches along which dispersal occurred by comparing the ranges with the highest probability between ancestral and descendent nodes. For each branch with a dispersal event, we randomly sampled a time for the event along the branch. In case of multiple source areas for a dispersal event, we used the time‐stratified matrix of areas allowed to narrow the possible sources down to only those permitted during each time period. If multiple sources were still possible, we sampled one of these randomly. We repeated this procedure 1000 times, and each time, we summed the number of transitions between all pairs of areas. The mean number of transitions was then calculated and represented using the R package qgraph (Epskamp et al., 2012).

### 2.6 Diversification dynamics

We are aware that these diversification models are controversial (see also Discussion part in Section 3), and while we recognize the shortcomings, we nevertheless wanted to see how multiple models compare with each other and think that it is still valuable to report these results. The hypothesis‐driven model selection framework used here is suitable for investigating speciation and extinction dynamics with appropriate assumptions (Helmstetter et al., 2021; Louca & Pennell, 2020).

We estimated the dynamics of speciation and extinction rates through time using three different birth‐death models to cross‐validate the results: First, we used the model TreePar (Stadler, 2011). The timing of divergence between the backbone and the *Lepidochrysops* was included in the backbone analysis. We isolated the *Lepidochrysops* clade from the rest of the tree (the ‘backbone’ throughout this paper) and modeled diversification for each partitioned tree independently. For each partition and 100 trees randomly sampled from the posterior distribution of our dating analysis, we fitted TreePar using fixed time bins of 4 My. Second, we used the model proposed by Morlon et al. (2011) and implemented in the R‐package RPANDA v.1.8 (Morlon et al., 2016). As above the phylogenetic tree was partitioned into the *Lepidochrysops* clade and the backbone. Speciation and extinction rates were modeled using both linear and exponential functions of time. For each partition of the tree, we fitted 12 models: constant speciation, no extinction; constant speciation, constant extinction; time‐dependent speciation (exponential or linear), no extinction; time‐dependent speciation (exponential or linear), constant extinction; constant speciation, time‐dependent extinction (exponential or linear); time‐dependent speciation (exponential or linear); and time‐dependent extinction (exponential or linear). For the backbone, the models accounted for the divergence event between the backbone and *Lepidochrysops*. However, the stem of the *Lepidochrysops* clade was included in the *Lepidochrysops* model of diversification, following the original implementation of the method. Sampling fractions were specified for each tree partition. Models were fitted on 100 trees randomly sampled from the posterior distribution of the dating analysis. Models were ranked according to their AIC scores averaged across the posterior distribution (Table S1). Third, the results of both the above methods indicated a consistent pattern of extinction around 10 Mya. Hence, we fitted the model CoMET (May et al., 2016) using the R‐package TESS v. 2.1.0 (Höhna et al., 2016) on the full tree (no partitioning) to assess the support for a tree‐wide extinction event. We performed a reversible‐jump MCMC analysis assuming constant speciation and extinction rates through time. We set the number of expected sudden extinctions to 1, with an expected survival probability of 0.1. We ran the rjMCMC for 10 million generations, removing the first 10,000 as burn‐in.

## 3 RESULTS AND DISCUSSION

### 3.1 Diversification during the aridification of Africa

We inferred a molecular phylogeny for 179 members of the *Euchrysops* section, plus seven outgroups, based on 419 loci obtained from anchored hybrid enrichment. Concatenation and summary coalescent approaches produced similar topologies (Figures S1 and S2). Our dated phylogeny including 124 species showed that the *Euchrysops* section originated in the early Miocene, around 22 Mya (ci: 19–24 Mya) (Figure 1, Figure S3), congruent with the onset of African environmental change. The phyto‐predaceous *Lepidochrysops* originated much later at around 6.4 Mya (ci: 5.5–7.5 Mya), a time characterized by increased aridification (Herbert et al., 2016).

### 3.2 Increasing occupation of drier biomes

We estimated that the *Euchrysops* section (Lycaenidae, Polyommatinae) likely originated in the emerging Miombo woodlands (Jacobs, 2004), now covering large parts of southern‐central Africa (Figure 1).

**FIGURE 2:**
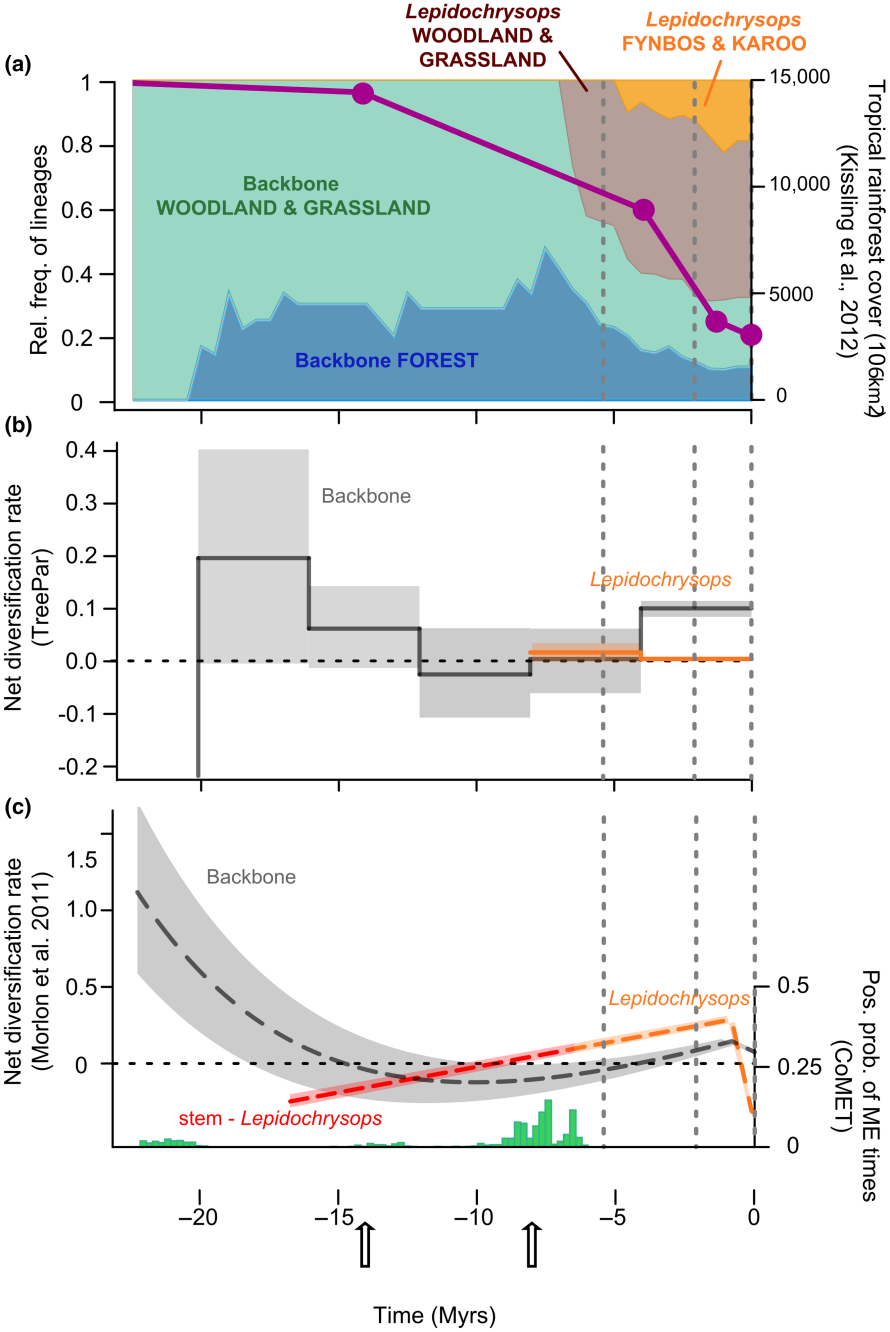
(a) Estimated relative frequency of lineages in different biomes through time (left *Y*‐axis). The purple line shows the reduction of rainforest cover (in 10^6^ km^2^) through time in Africa, based on data from Kissling et al. (2012) (right *Y*‐axis). (b) Net diversification rate for *Lepidochrysops* and the backbone using TreePar. (c) Net diversification rate according to the best models identified for *Lepidochrysops* and the backbone using Morlon et al. (2011) (left *Y*‐axis). The histogram shows the posterior probabilities of a sudden extinction event through time, estimated using the model CoMET (right *Y*‐axis). Black arrows at the bottom denote the Middle Miocene Cooling event (MMCO) and the Late Miocene Cooling event (LMCO), respectively.

Biome occupation was characterized by extensive turnover during the last 7 My (Figure 2a). Between 20 and 8 Mya, ~30% of lineages occupied forests. With the diversification of *Lepidochrysops*, this fraction decreased to 10% in the last 8 My, coinciding with the dramatically decreasing rainforest cover in Africa during the Miocene (Kissling et al., 2012) (Figure 2a). No extant mainland‐Africa *Lepidochrysops* currently inhabit forests (a few species occur in forest‐savanna transition). The only forest *Lepidochrysops* are found in Madagascar where five species occur in dry spiny forest or rainforest, only one of which could be included in this study (Figure S1). We excluded this one from the biogeographical analyses in order to reduce the number of areas in the analysis. This should not have a strong impact on our analyses since these Malagasy species form a single, small radiation (Espeland, in preparation).

**FIGURE 3:**
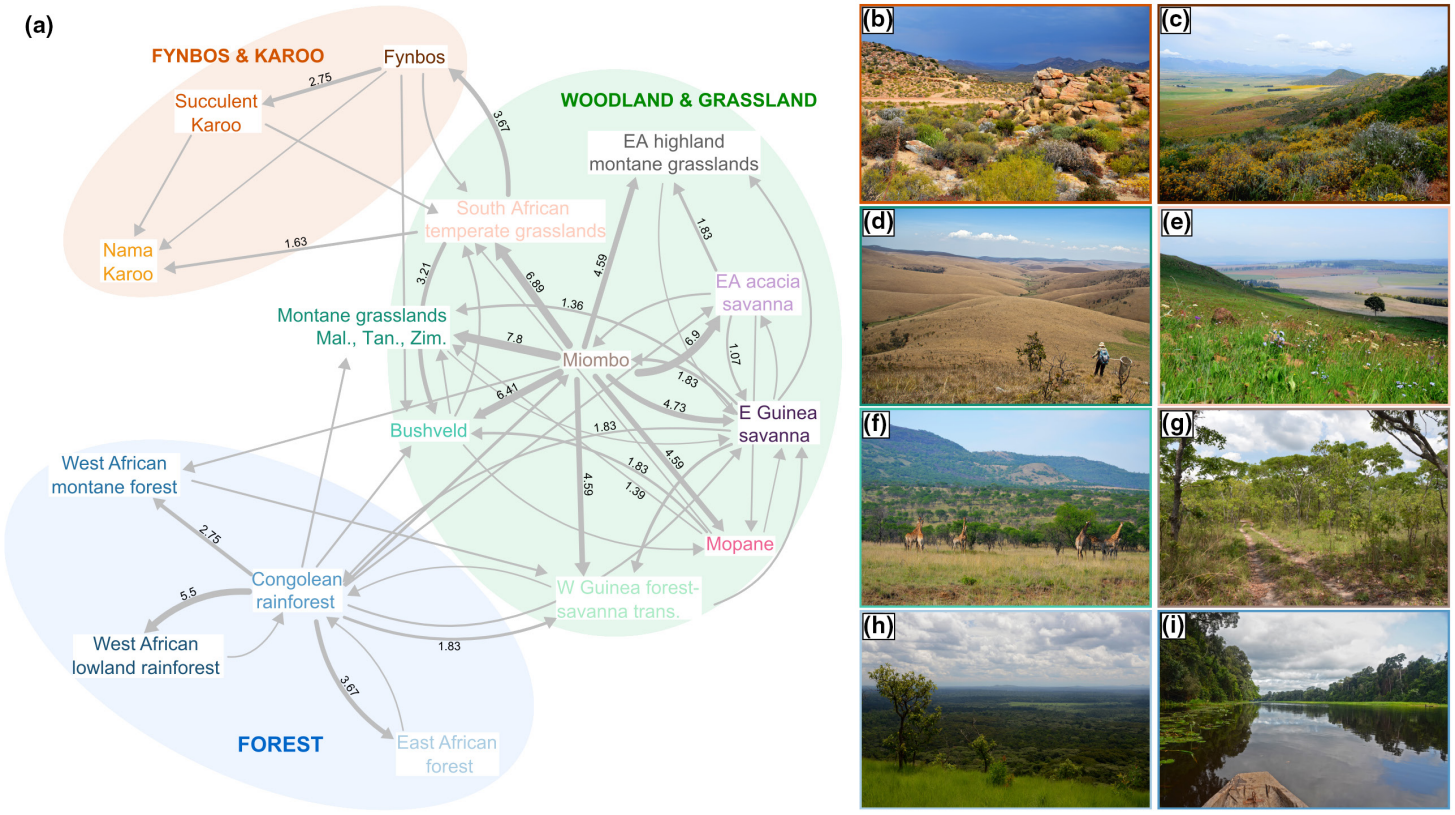
(a) Sum of transitions between biomes by members of the *Euchrysops* section and their ancestors as estimated from biogeographical analyses. Thicker arrows/higher numbers indicate more transitions between biomes. E, Eastern; EA, East African; Mal., Malawi; Tan., Tanzania; trans., transition zone; W, Western; Zim., Zimbabwe. Photos of the biomes include (b) Succulent Karoo, (c) Fynbos, (d) Malawian, Zimbabwean, southern Tanzanian montane grasslands, (e) Southern African montane grasslands, (f) Bushveld, (g) Miombo woodland, (h) East African forest, and (i) Congolean rainforest. All photos by M. Espeland.

Miombo woodlands played a key role as the source for many dispersal events toward other woodland and grassland biomes, both for the clade as a whole (Figure 3) and for *Lepidochrysops* alone (Figure S4). Adding additional outgroups would not change this result, since the most closely related clades are largely non‐African (Tonini et al., in prep.). Likewise, South African temperate grasslands constituted an important stepping‐stone toward the more arid Fynbos and Karoo biomes for the phyto‐predaceous *Lepidochrysops* lineages. These arid biomes were not reached by any phytophagous lineages in the clade, with the exception of the genus *Orachrysops*, where two species are found in wetter parts of coastal and montane Fynbos, but the remaining nine species exclusively occur in the South African montane grasslands. *Orachrysops* are phytophagous (feeding on *Indigofera*, Fabaceae) and the larvae are either facultatively or obligately ant associated. The larvae of several species feed on the rootstocks of *Indigofera* from the third instar onwards, tended by *Camponotus* ants, and thus spend most of their time underground tightly associated with ants (Edge & Van Hamburg, 2010; Lu & Samways, 2001). Interestingly, this genus, previously placed in *Lepidochrysops*, is sister to the rest of the section, and appears to have diversified within the last 5 My, coincident with the origin of the grassland biome (Jacobs et al., 1999).

Little dispersal has taken place between forests and drier biomes (Figure 3). The forest lineages *Oboronia* and *Thermoniphas* are sister groups, whose ancestor likely originated in the Congolian rainforest after dispersing from the Miombo woodlands. *Oboronia* feeds exclusively on *Costus* (Zingiberaceae) and is obligately mutualistic with *Pheidole* ants (Lamborn, 1914; Sourakov & Emmel, 1997). Little is known about the life history of *Thermoniphas*, but it is thought that ant association is limited, and one species is known to feed on Melastomataceae (Congdon et al., 2017; Heath et al., 2002).

*Euchrysops* is polyphyletic and consists of at least four different clades: the *cnejus*, *barkeri*, *albistriata*, and *dolorosa* groups (Figure S1). All four groups likely originated in the Miombo woodlands, although *E. barkeri*, which is sister to *Oboronia* and *Thermoniphas* (Figure S1), might have originated in the forest before dispersing back to the Miombo woodlands and subsequently becoming widespread but localized across savannas and woodlands. The *E. dolorosa* group is sister to *Lepidochrysops*, and widespread, occurring in most biomes except forests, Fynbos and Karoo. *E. dolorosa* group members largely feed on *Ocimum* (Lamiaceae), which is also used by many *Lepidochrysops* species, and have an apparently mutualistic association with ants (e.g. Larsen, 2005, personal observation ME), indicating a transition from mutualism to parasitism in the ancestor of *Lepidochrysops*, but no host‐plant shift.

The genus *Lepidochrysops* is monophyletic. Although the group diverged more than 15 Mya (Figure 1), the last common ancestor of the extant lineages is only ~6.4 My old. Parasitism of ant associates arose along the 10 My‐long stem branch, likely just prior to *Lepidochrysops*' rapid radiation. The genus consists of two major clades: one originated in the Southern African montane grasslands and dispersed from there to the Fynbos and Karoo biomes. The other originated further north, likely in the Miombo woodlands, and from there spread throughout the woodland and grassland biomes. Interestingly, a few species also in this clade reached the Southern African montane grasslands, before a single dispersal to the Fynbos, further underscoring the importance of these grasslands as a source of phyto‐predaceous taxa in the summer arid biomes.

### 3.3 Extinction and rise of phyto‐predation

In TreePar analyses, the backbone showed a pattern of decreasing net diversification from the root, reaching its minimum around 10 Mya and increasing again towards the present. For the *Lepidochrysops* (stem branch excluded; Figure 2b) we estimated an overall constant positive net diversification rate.

Using the Morlon et al. model for the backbone, we found three models falling within an AIC interval of two (Table S1A–C), and all inferred a decline of diversity. The model with speciation and extinction as exponential and linear functions of time, respectively, was remarkably similar to the results from TreePar (Figure 2C). Net diversification declined rapidly, reaching a minimum around 10 Mya, before increasing again towards the present. Negative net diversification rates between 15 and 5 Mya indicated declining diversity. The other two models suggested either a global decrease of diversification through time with a negative net diversification rate during the last 8 My, or an earlier decline with a negative net diversification rate for the last 15 My. In *Lepidochrysops* (including stem branch) the best model corresponded to linear speciation and extinction functions of time (Table S1D–F). Net diversification increased through time but remained negative along the stem of the *Lepidochrysops* clade until ~10 Mya (Figure 2C). This result is unsurprising considering the length of the stem (10.2 My), which most likely results from past extinction events. Net diversification was positive during the last 10 My.

Finally, CoMET (Figure 2c) found a signal of tree‐wide extinction around 6–8 Mya, with moderate support. The timing matches well with the pattern of declining diversity identified by both TreePar and Morlon et al. analyses during the late Miocene. It also coincides with the late Miocene cooling event, a time with increased aridification and a marked decrease in temperature (Herbert et al., 2016).

Our hypothesis that the Miocene aridification of Africa strongly influenced the *Euchrysops* section is supported by the consistent signal of extinction in the phytophagous backbone lineages during the middle‐ to late Miocene, and the rapid radiation of the phyto‐predaceous *Lepidochrysops* lineages shortly thereafter. Our repeated finding of a pattern of extinction suggests that our phylogeny does carry some signal of extinction. Estimating extinction rates or diversity decline from phylogenies without fossils is, however, challenging and controversial (Beaulieu & O'Meara, 2015; Burin et al., 2019; Louca & Pennell, 2020; Morlon, 2014; Nee et al., 1994; Paradis, 2004), and phylogenetic studies have been criticized for showing low extinction rates compared with the fossil record (Burin et al., 2019; Louca & Pennell, 2020; Paradis, 2004; Quental & Marshall, 2011). This may result from use of inappropriate methods that preclude negative diversification estimates (Magallon & Sanderson, 2001; Rabosky, 2006), failure to properly consider time and clade heterogeneity (Morlon, 2014) or problems inherent to the models themselves (Louca & Pennell, 2020). Unfortunately, in groups where fossil information is largely unavailable, like butterflies, birth‐death models currently provide the only resource available to assess past diversification.

Another important caveat is that our analysis is based only on lineages included in our phylogeny, which are necessarily extant taxa. High levels of extinction between 15 and 5 Mya, may well be obscuring the actual number of lineages during this period. If forest taxa were the most affected by extinctions as their habitats retreated, it is possible that forest biome lineages represented an even greater proportion of the diversity in the past.

### 3.4 Aphytophagy: A rare and risky life history strategy in Lepidoptera

The success of phyto‐predation in the *Lepidochrysops* radiation is highly unusual: over 99% of Lepidoptera caterpillars are exclusively phytophagous (Pierce, 1995). The few butterfly species feeding on resources other than plants (i.e., aphytophagous lineages) are found primarily in Lycaenidae and Riodinidae, which have independently evolved high degrees of ant association (Espeland et al., 2018; Pierce et al., 2002). Symbiotic ant association is thought to have promoted diversification in the Lycaenidae as a whole (Pellissier et al., 2017; Pierce et al., 2002; Schär et al., 2018). However, even though entomophagy has evolved many times independently in Lycaenidae, this strategy generally appears to be an evolutionary dead‐end since it has rarely led to diversification (Cottrell, 1984; Pierce, 1995; Pierce et al., 2002; Schär et al., 2018), with *Lepidochrysops* as the striking exception.

For *Lepidochrysops*, our results suggest that harsh conditions above ground, such as fire or long dry seasons, favored shifts into ant nests and contributed to the evolutionary success of phyto‐predation (Cottrell, 1984; Fiedler, 1991, 1998). This hypothesis parallels findings that burrowing mammals better cope with climate change in arid environments than animals that do not live underground (Riddell et al., 2021), and that plants in fire‐prone savannas repeatedly evolve underground life forms (Maurin et al., 2014).

Consistent with aphytophagy being evolutionarily precarious, it is over‐represented among species with threatened conservation status on the IUCN Red List of Threatened Species and accounts for approximately 15% of the butterflies listed in the highest categories (IUCN, 2020), despite making up considerably less than 1% of butterfly species overall. Even in *Lepidochrysops*, which have adaptations to survive climate change, most of the assessed species are listed as endangered to rare, one is recorded as extinct, and others are thought to be extinct (Mecenero et al., 2020).

### 3.5 Ant association and adaptation to the aridification of Africa

Few studies have investigated the influence of the Miocene aridification (but see Aduse‐Poku et al., 2021; Kergoat et al., 2018). Several of the few notable radiations in drier areas are, however, associated with ants. In addition to *Lepidochrysops*, these include the ant‐parasitic *Paussus* beetles (>350 species) (Moore & Robertson, 2014), and ant‐associated butterflies in the lycaenid genera *Chrysoritis*, *Aloeides*, and *Thestor*, with a moderate number of lineages (27–57 described species) found mainly in southern Africa (Heath et al., 2023; Quek et al., 2022; Rand et al., 2000; Talavera et al., 2020).

Larvae of *Chrysoritis* and *Aloeides* are strongly ant associated and belong to the Aphnaeinae, a largely African subfamily with about 300 species, where ant parasitism has evolved multiple times independently (Boyle et al., 2015; Pierce et al., 2002). Potential adaptations to life in arid biomes thus most likely evolved after they arrived in these areas. *Chrysoritis* occurs within a variety of biomes from semi‐desert to forest (Talavera et al., 2020) in southern Africa, originating around 17 Mya but, like *Lepidochrysops*, only radiating rapidly in the Fynbos and the Succulent Karoo within the last 2.5 My (Talavera et al., 2020). The genera *Aloeides* and *Thestor* both arose between 5 and 10 My and have undergone limited radiation only in the GCFR (Boyle et al., 2015; Kaliszewska et al., 2015). Differently from *Chrysoritis* and *Aloeides*, *Lepidochrysops* species were already phyto‐predaceous when they reached the Fynbos and Karoo and were thus pre‐adapted to these arid environments. This is also the case for *Thestor* species, since all species in the subfamily Miletinae are aphytophagous where known, parasitizing either ants or ant‐associated homopterans (Kaliszewska et al., 2015).

## 4 CONCLUSIONS

We show that phytophagous lineages in the *Euchrysops* section of the butterfly family Lycaenidae experienced high levels of extinction during the aridification of Africa between 15 and 5 Mya. In contrast, the radiation of the aphytophagous genus *Lepidochrysops* originated around 6.5 Mya, shortly after aphytophagy evolved. The Miombo woodlands were likely the cradle for diversification of the *Euchrysops* section, and our findings are consistent with the hypothesis that aridification during the Miocene selected for a phyto‐predaceous life history, with ant nests providing caterpillars a safe refuge from fire and a source of food when vegetation was scarce. Reproductive diapause during the dry season is thought to be another important adaptation for the survival of butterflies in African savannas (Halali et al., 2020). Phyto‐predation can be seen as an even more extreme strategy to avoid unfavorable conditions, and seemingly facilitated radiations even in more arid biomes. Penetration of ant nests must have required the evolution of a suite of pheromones and behaviors to mimic and manipulate ant hosts, of which little has yet been learned for this group. Since phyto‐predaceous behavior in *Lepidochrysops* and *Phengaris* evolved independently, a genomic comparison of these convergent systems could help illuminate constraints and contingencies influencing the evolution of these iconic butterflies.

## AUTHOR CONTRIBUTIONS

**Marianne Espeland:** Conceptualization (equal); formal analysis (lead); funding acquisition (lead); investigation (lead); methodology (lead); project administration (lead); resources (equal); supervision (equal); visualization (lead); writing – original draft (lead); writing – review and editing (lead). **Nicolas Chazot:** Formal analysis (equal); investigation (equal); methodology (equal); visualization (supporting); writing – original draft (supporting); writing – review and editing (supporting). **Fabien L. Condamine:** Formal analysis (equal); methodology (equal); visualization (supporting); writing – original draft (supporting); writing – review and editing (supporting). **Alan R. Lemmon:** Formal analysis (supporting); methodology (supporting); writing – original draft (supporting); writing – review and editing (supporting). **Emily Moriarty Lemmon:** Formal analysis (supporting); methodology (supporting); writing – original draft (supporting); writing – review and editing (supporting). **Ernest Pringle:** Investigation (supporting); resources (supporting); writing – original draft (supporting); writing – review and editing (supporting). **Alan Heath:** Resources (equal); writing – original draft (supporting); writing – review and editing (supporting). **Steve Collins:** Resources (supporting); writing – original draft (supporting); writing – review and editing (supporting). **Wilson Tiren:** Resources (supporting); writing – original draft (supporting); writing – review and editing (supporting). **Martha Mutiso:** Resources (supporting); writing – original draft (supporting); writing – review and editing (supporting). **David C. Lees:** Resources (supporting); writing – original draft (supporting); writing – review and editing (supporting). **Stewart Fisher:** Resources (supporting); writing – original draft (supporting); writing – review and editing (supporting). **Raymond Murphy:** Resources (supporting); writing – original draft (supporting); writing – review and editing (supporting). **Stephen Woodhall:** Resources (supporting); writing – original draft (supporting); writing – review and editing (supporting). **Robert Tropek:** Resources (supporting); writing – original draft (supporting); writing – review and editing (supporting). **Svenja S. Ahlborn:** Data curation (supporting); investigation (supporting); writing – review and editing (supporting). **Kevin Cockburn:** Resources (supporting); writing – original draft (supporting); writing – review and editing (supporting). **Jeremy Dobson:** Resources (supporting); writing – original draft (supporting); writing – review and editing (supporting). **Thierry Bouyer:** Resources (supporting); writing – original draft (supporting); writing – review and editing (supporting). **Zofia A. Kaliszewska:** Resources (supporting); writing – original draft (supporting); writing – review and editing (supporting). **Christopher C. M. Baker:** Data curation (supporting); formal analysis (supporting); writing – original draft (equal); writing – review and editing (supporting). **Gerard Talavera:** Resources (supporting); writing – original draft (supporting); writing – review and editing (supporting). **Roger Vila:** Investigation (supporting); resources (supporting); writing – original draft (supporting); writing – review and editing (supporting). **Alan J. Gardiner:** Investigation (supporting); resources (supporting); writing – original draft (supporting); writing – review and editing (supporting). **Mark Williams:** Investigation (supporting); resources (supporting); writing – original draft (supporting); writing – review and editing (supporting). **Dino J. Martins:** Investigation (supporting); resources (supporting); writing – original draft (supporting); writing – review and editing (supporting). **Szabolcs Sáfián:** Investigation (supporting); resources (supporting); writing – original draft (supporting); writing – review and editing (supporting). **David A. Edge:** Investigation (supporting); resources (equal); writing – original draft (supporting); writing – review and editing (supporting). **Naomi E. Pierce:** Conceptualization (equal); funding acquisition (equal); investigation (supporting); project administration (equal); supervision (equal); writing – original draft (equal); writing – review and editing (equal).

## CONFLICT OF INTEREST STATEMENT

None declared.

## Supporting information


Data S1.

Figure S1.

Figure S2.

Figure S3.

Figure S4.

Table S1.

## Data Availability

Data supporting the study are available in the main text or the supplementary information. Raw reads for all samples have been deposited in the NCBI Sequence Read Archive, Bioproject PRJNA892447, accession numbers SRR24203818 to SRR24203998. Alignments, tree files and selected models and partitions are available on Zenodo (DOI: 10.5281/zenodo.4590738).
