# Supplementary material for: Rapid radiation of ant parasitic butterflies during the Miocene aridification of Africa
Source: Ecol Evol. 2023 May 13;13(5):e10046. doi: 10.1002/ece3.10046 (PMC10182571; doi:10.1002/ece3.10046)
Supplement: Supplementary file 2 — Figure S1. [file ECE3-13-e10046-s001.pdf]

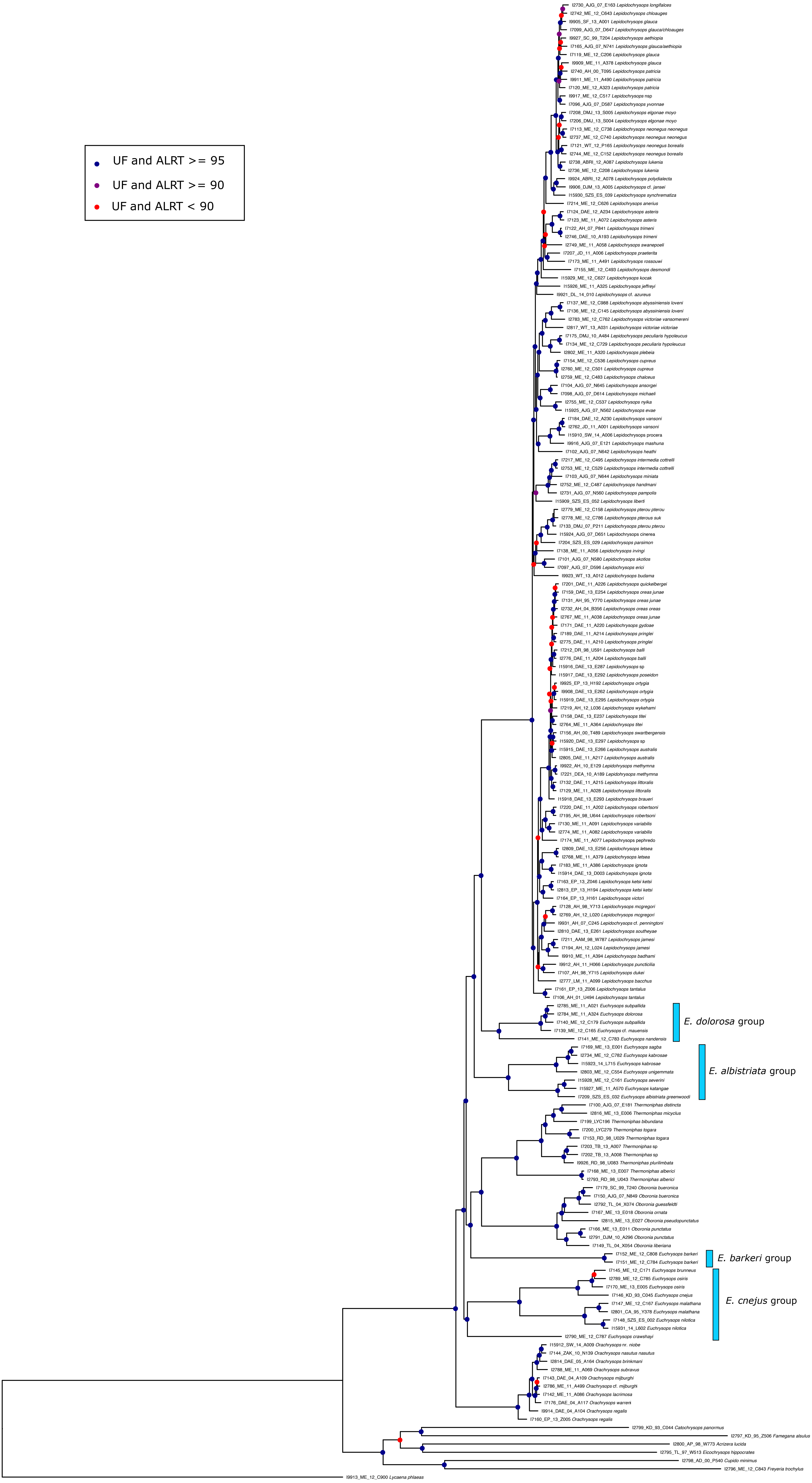

Figure S1. Full phylogeny inferred using a concatenaton approach in IQ-TREE. UF = ultrafast bootstrap support, ALRT = SH-like approximate likelihood ratio test branch support
