# Supplementary material for: Rapid radiation of ant parasitic butterflies during the Miocene aridification of Africa
Source: Ecol Evol. 2023 May 13;13(5):e10046. doi: 10.1002/ece3.10046 (PMC10182571; doi:10.1002/ece3.10046)
Supplement: Supplementary file 3 — Figure S2. [file ECE3-13-e10046-s002.pdf]

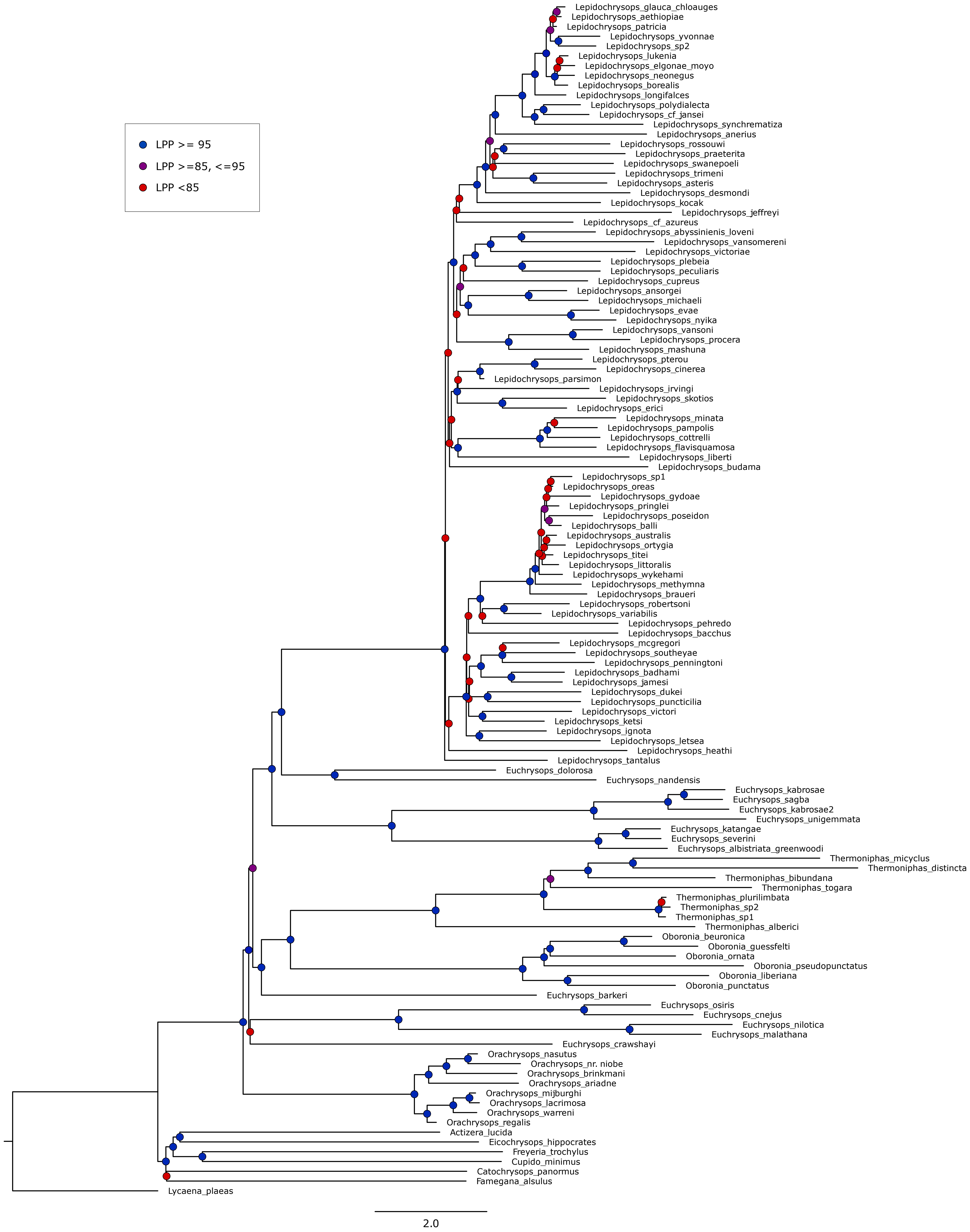

Figure S2. Species tree inferred using ASTRAL-III. Branch support is given as local posterior probabilities. Branch lengths are in coalescent units.
