# Supplementary material for: Rapid radiation of ant parasitic butterflies during the Miocene aridification of Africa
Source: Ecol Evol. 2023 May 13;13(5):e10046. doi: 10.1002/ece3.10046 (PMC10182571; doi:10.1002/ece3.10046)
Supplement: Supplementary file 4 — Figure S3. [file ECE3-13-e10046-s005.pdf]

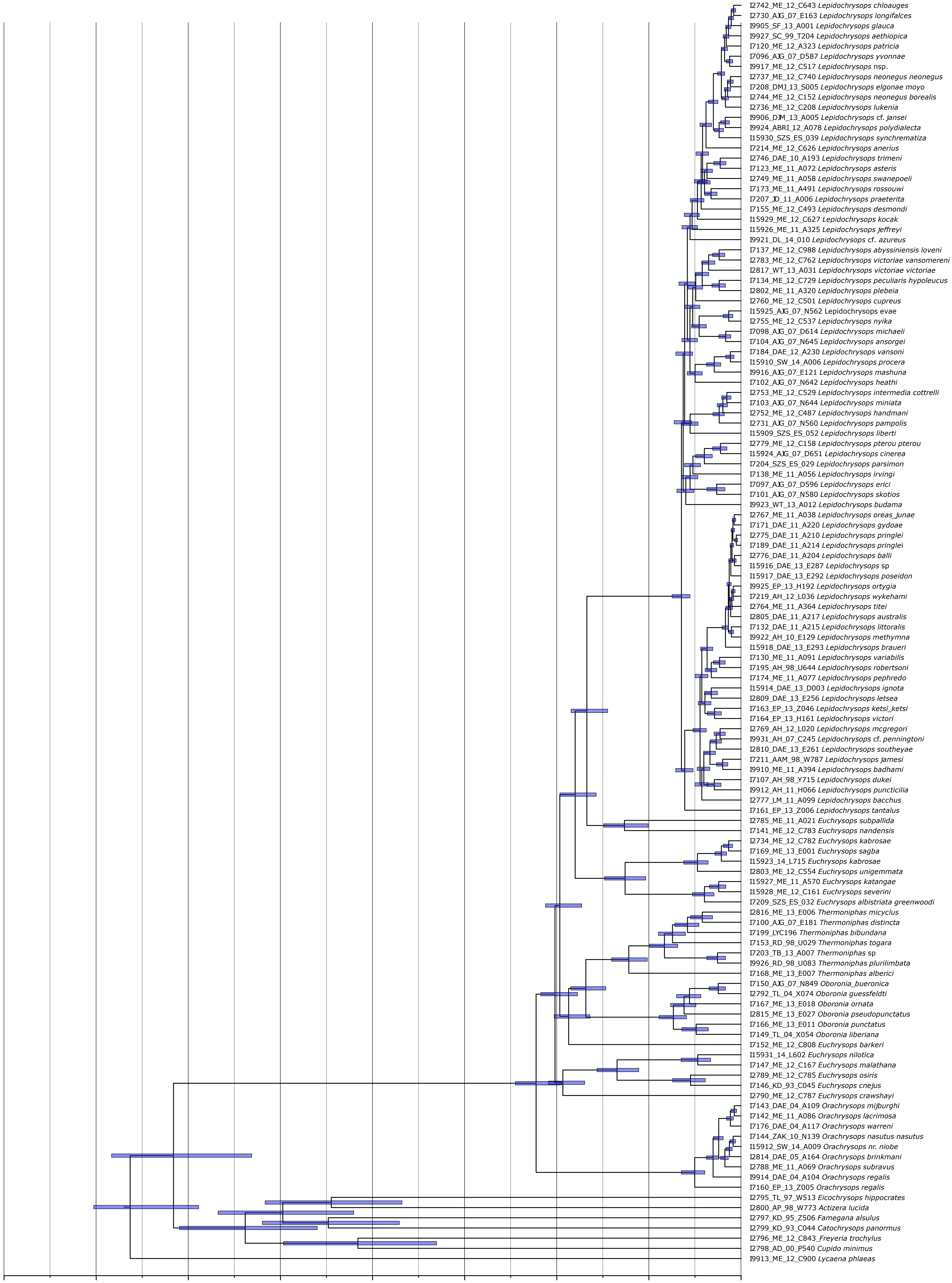

Figure S3. Dated phylogeny (MCMCtree) based on reduced dataset only including one specimen per species. Blue bars show the 95% credibility intervals.
