# Supplementary material for: Rapid radiation of ant parasitic butterflies during the Miocene aridification of Africa
Source: Ecol Evol. 2023 May 13;13(5):e10046. doi: 10.1002/ece3.10046 (PMC10182571; doi:10.1002/ece3.10046)
Supplement: Supplementary file 5 — Figure S4. [file ECE3-13-e10046-s006.pdf]

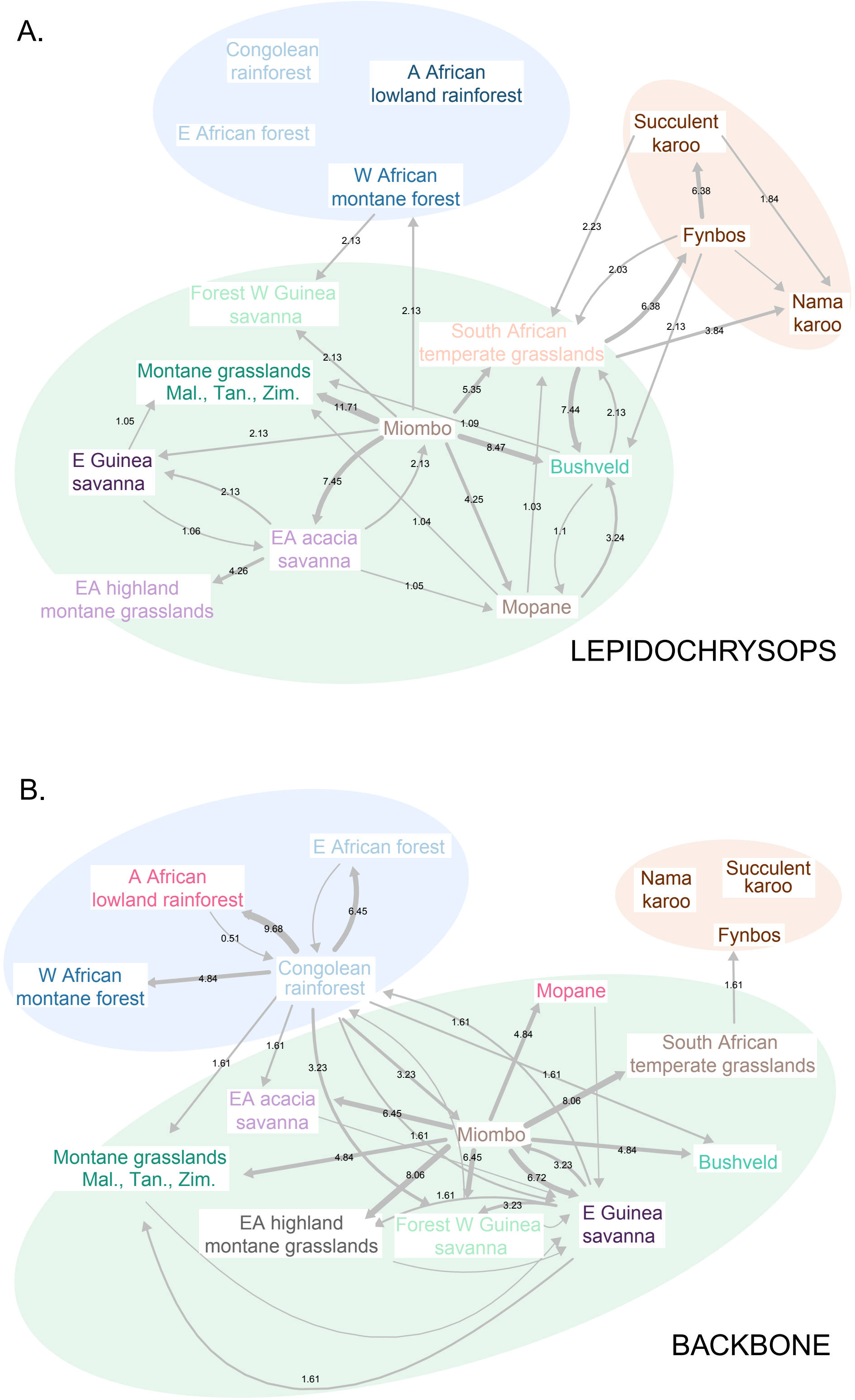

Figure S4. Sum of transitions between biomes made by members of a) *Lepidochrysops* and their ancestors as estimated from biogeographical analyses and b) the remainder of the backbone (excluding *Lepidochrysops)*. Thicker arrows/higher numbers indicate more transitions between biomes. EA = East African, E = Eastern, W = Western, Mal. = Malawi, Zim. = Zimbabwe, Tan. = Tanzania.
