# Supplementary material for: Rapid radiation of ant parasitic butterflies during the Miocene aridification of Africa
Source: Ecol Evol. 2023 May 13;13(5):e10046. doi: 10.1002/ece3.10046 (PMC10182571; doi:10.1002/ece3.10046)
Supplement: Supplementary file 6 — Table S1. [file ECE3-13-e10046-s003.docx]

Table S1: **Results from the Morlon et al. (2011) models.**  BCST = constant speciation rate, DCST = constant extinction rate, BVAR = time-dependent speciation rate, DVAR = time-dependent extinction rate, Bexpo = exponential time-dependency of speciation rate, Dlin = linear time-dependency of extinction rate, Dexpo = exponential time-dependency of extinction rate, logL = likelihood, AIC = Akaike information criterion, λ = speciation rate at present, ⍺ = coefficient of speciation rate time variation, μ = extinction rate at present, β = coefficient of extinction rate time variation. A) Models fitted on the backbone with exponential time-dependency of speciation and/or extinction rates.

| **Model** | **Num.par** | **logL** | **AIC** | **λ** | **⍺** | **μ** | **β** |
| --- | --- | --- | --- | --- | --- | --- | --- |
| BCST | 1 | -112.91 (1.91) | 227.83 (3.82) | 0.136 (0.007) |  |  |  |
| BCST / DCST | 2 | -112.73 (1.92) | 229.47 (3.85) | 0.160 (0.014) |  | 0.043 (0.019) |  |
| BVAR | 2 | -112.86 (1.91) | 229.73 (3.82) | 0.130 (0.009) | 0.006 (0.006) |  |  |
| BVAR / DCST | 3 | -111.39 (1.97) | 228.79 (3.95) | 0.200 (0.020) | 0.050 (0.005) | 0.257 (0.042) |  |
| BCST / DVAR | 3 | -112.55 (1.94) | 231.11 (3.88) | 0.188 (0.024) |  | 0.139 (0.057) | -0.072 (0.023) |
| BVAR / DVAR | 4 | -108.41 (2.23) | 224.83 (4.46) | 0.150 (0.017) | 0.205 (0.023) | 0.197 (0.025) | 0.189 (0.022) |

B) models fitted on the backbone with linear time-dependency of speciation and/or extinction rates,

| **Model** | **Num.par** | **logL** | **AIC** | **λ** | **⍺** | **μ** | **β** |
| --- | --- | --- | --- | --- | --- | --- | --- |
| BCST | 1 | -112.91 (1.91) | 227.83 (3.82) | 0.136 (0.007) |  |  |  |
| BCST / DCST | 2 | -112.73 (1.92) | 229.47 (3.85) | 0.160 (0.014) |  | 0.043 (0.019) |  |
| BVAR | 2 | -112.87 (1.91) | 229.75 (3.82) | 0.131 (0.009) | 7E-04 (7E-04) |  |  |
| BVAR / DCST | 3 | -111.42 (1.96) | 228.84 (3.92) | 0.193 (0.021) | 0.018 (0.003) | 0.293 (0.043) |  |
| BCST / DVAR | 3 | -112.34 (1.95) | 230.68 (3.91) | 0.204 (0.025) |  | 0.181 (0.055) | -0.009 (0.002) |
| BVAR / DVAR | 4 | -106.52 (3.45) | 221.04 (6.90) | -0.100 (0.039) | 0.336 (0.132) | 0.353 (0.082) | 0.175 (0.271) |

C) models fitted on the backbone with exponential and linear time-dependency of speciation and extinction rates,

| **Model** | **Num.par** | **logL** | **AIC** | **λ** | **⍺** | **μ** | **β** |
| --- | --- | --- | --- | --- | --- | --- | --- |
| Blin / Dexpo | 4 | -110.45 (1.93) | 222.91 (3.86) | 0.143 (0.030) | 0.073 (0.073) | 0.435 (0.039) | 0.061 (0.007) |
| Bexpo / Dlin | 4 | -108.83 (2.24) | 221.67 (4.48) | 0.130 (0.013) | 0.134 (0.014) | 0.056 (0.047) | -0.068 (0.015) |

D) models fitted on the *Lepidochrysops* clade with exponential time-dependency of speciation and/or extinction rates,

| **Model** | **Num.par** | **logL** | **AIC** | **λ** | **⍺** | **μ** | **β** |
| --- | --- | --- | --- | --- | --- | --- | --- |
| BCST | 1 | -167.64 (5.38) | 337.28 (10.76) | 0.410 (0.029) |  |  |  |
| BCST / DCST | 2 | -167.64 (5.38) | 339.28 (10.76) | 0.410 (0.029) |  | 1.11E-07 (8.9E-08) |  |
| BVAR | 2 | -167.09 (5.26) | 338.19 (0.034) | 0.371 (0.034) | 0.038 (0.012) |  |  |
| BVAR / DCST | 3 | -167.09 (5.26) | 340.19 (0.034) | 0.371 (0.034) | 0.038 (0.12) | 7.15E-08 (5.7E-08) |  |
| BCST / DVAR | 3 | -167.64 (5.38) | 341.28 (0.029) | 0.410 (0.029) |  | 7.68E-08 (7.1E-08) | 0.034 (0.019) |
| BVAR / DVAR | 4 | -167.09 (5.26) | 342.19 (0.034) | 0.371 (0.034) | 0.038 (0.012) | 5.98E-08 (4.8E-08) | 0.067 (0.022) |

E) models fitted on the *Lepidochrysops* clade with linear time-dependency of speciation and/or extinction rates,

| **Model** | **Num.par** | **logL** | **AIC** | **λ** | **⍺** | **μ** | **β** |
| --- | --- | --- | --- | --- | --- | --- | --- |
| BCST | 1 | -167.64 (5.38) | 337.28 (10.76) | 0.409 (0.028) |  |  |  |
| BCST / DCST | 2 | -167.64 (5.38) | 339.28 (10.76) | 0.409 (0.028) |  | 1.11E-07 (8.90E-08) |  |
| BVAR | 2 | -166.25 (5.14) | 336.50 (10.29) | 0.299 (0.043) | 0.043 (0.012) |  |  |
| BVAR / DCST | 3 | -166.25 (5.14) | 338.50 (10.29) | 0.299 (0.043) | 0.043 (0.012) | 8.03E-08 (7.91E-08) |  |
| BCST / DVAR | 3 | -167.64 (5.38) | 341.28 (10.76) | 0.409 (0.028) |  | 7.99E-08 (8.02E-08) | 0.012 (0.013) |
| BVAR / DVAR | 4 | -157.59 (5.91) | 323.19 (11.83) | -0.045 (0.029) | 0.399 (0.071) | -0.361 (0.091) | 0.432 (0.074) |

F) models fitted on the *Lepidochrysops* clade with exponential and linear time-dependency of speciation and extinction rates.

| **Model** | **Num.par** | **logL** | **AIC** | **λ** | **⍺** | **μ** | **β** |
| --- | --- | --- | --- | --- | --- | --- | --- |
| Blin / Dexpo | 4 | -165.88 (5.24) | 333.76 (10.49) | 0.292 (0.060) | 0.053 (0.056) | 0.014 (0.091) | -0.433 (1.051) |
| Bexpo / Dlin | 4 | -164.03 (5.66) | 332.07 (11.33) | 0.479 (0.051) | 0.099 (0.010) | 0.605 (0.150) | -0.196 (0.050) |
